# Reassessing Dementia Risk: Expanding the 2024 Lancet Commission Report for a More Accurate Global Understanding

**DOI:** 10.1002/alz70858_098514

**Published:** 2025-12-25

**Authors:** Cyprian M Mostert

**Affiliations:** ^1^ Global Brain Health Institute, Trinity College Dublin, University of Dublin, Ireland

## Abstract

**Background:**

The 2024 Lancet Commission Report analyzes 14 risk factors that account for 45 percent of global dementia cases. However, it overlooks four critical risk factors: poverty, wealth shocks, income inequality, and viral infections. This is particularly concerning given that 57 percent of the identified risk factors in the Lancet report are more prevalent in men, even though dementia predominantly affects women worldwide.

**Method:**

In this policy paper, we used the recent global review studies and argue that it is essential to recalibrate and expand the 2024 Lancet Commission's list of risk factors to include poverty, wealth shocks, income inequality, and viral infections. Our analysis suggests that these overlooked risk factors could add an additional 20 percent to the risk of developing dementia, indicating that up to 65 percent of global dementia cases might be preventable.

**Result:**

Furthermore, recognizing these additional risk factors allows for a more accurate representation of the global gender disparities in dementia progression. By incorporating these elements, we demonstrate that 55 percent of global dementia cases are more prevalent in women than in men, aligning with the true epidemiological patterns observed worldwide.

**Conclusion:**

There is a necessity to calibrate the risk factors of the 2024 lancet commission to improve its global applicability.

